# Real-World Evidence: Cefiderocol Therapeutic Drug Monitoring in Critically Ill, Obese Patients with *Klebsiella pneumoniae* Infections

**DOI:** 10.3390/antibiotics15060619

**Published:** 2026-06-18

**Authors:** Alessandra Manca, Alice Palermiti, Silvia Corcione, Giorgia Menegatti, Jessica Cusato, Cecilia Grosso, Chiara Risso, Giorgia Giuseppina Montrucchio, Silvia Scabini, Filippo Mariano, Amedeo De Nicolò, Francesco Giuseppe De Rosa, Antonio D’Avolio

**Affiliations:** 1 Laboratory of Clinical Pharmacology and Pharmacogenetics, Department of Medical Sciences, Amedeo di Savoia Hospital, University of Turin, Corso Svizzera 164, 10149 Turin, Italy; 2 Department of Medical Sciences, Infectious Disease, University of Turin, City of Health and Science Hospital, 10126 Turin, Italy; 3 Tufts University School of Medicine, Boston, MA 02111, USA; 4 Department of Surgical Science, University of Turin, 10124 Turin, Italy; 5 Department of Anesthesia, Intensive Care and Emergency, City of Health and Science Hospital, 10126 Turin, Italy; 6 Department of Medical Sciences, Infectious Diseases, University of Turin, 10124 Turin, Italy; 7 Nephrology, Dialysis and Transplantation U, Department of Medical Sciences, University of Turin, City of Health and Science Hospital, 10126 Turin, Italy

**Keywords:** PK/PD target, FDC, new β-lactams, critically ill patients, obesity, ARC

## Abstract

**Background/Objectives:** Cefiderocol (FDC) is a siderophore-containing cephalosporin that retains activity against many β-lactamase-producing bacteria, such as New Delhi metallo-β-latamase-producing (NDM) *K. pneumoniae*. Its use in critically ill patients is still limited, since the recommended dosing regimens are mainly derived from studies on healthy subjects, while critical illness is often associated with critical alterations in drug pharmacokinetics. Therefore, the aim of this study was to investigate FDC pharmacokinetic/pharmacodynamic (PK/PD) parameters in real-life patients based on their body weight and renal function. **Methods:** Patients with *K. pneumoniae* infections and indications for FDC were enrolled. Drug quantification in plasma was performed at the steady state at different timings. PK/PD targets of *f*Cmin > 4 mg/L (most common) and more stringent targets of *f*Cmin > 8 and 12 mg/L (4× and 6× the EUCAST breakpoint MIC) were considered in relation to patients’ characteristics, 14 days of microbiological eradication and 30-day mortality. **Results:** Ten patients were enrolled in this study. Mortality, as well as the failure to achieve microbiological eradication, increased with BMI. In a PK/PD point of view, all patients reached the PK/PD targets of *f*Cmin > 4 mg/L and > 8 mg/L, while only 20% reached a *f*Cmin > 12 mg/L, with a key influence of renal function. However, no significant association was found between PK/PD target attainment and treatment outcomes. **Conclusions:** Our study may be useful for the real-world use of FDC, highlighting the impact of renal function on the achievement of ideal PK/PD thresholds. Nevertheless, the lack of a significant association between PK/PD and outcomes, partially due to the small sample size, highlights the complex impact of patients’ clinical conditions other than drug PK.

## 1. Introduction

Cefiderocol (FDC) is a new siderophore cephalosporin that is highly effective for Gram-negative bacilli, including carbapenemase-producing and some metal-β-lactamase-producing forms of Enterobacterales [1]. Among the clinical trials that have explored the efficacy of FDC, CREDIBLE-CR focused on ventilator-associated and hospital-acquired pneumonia (VAP and HAP, respectively) [2]. This study highlighted the noninferiority of FDC to the standard of care in terms of microbial eradication, even though a numerically higher mortality was observed in the FDC arm, suspected to be due to differences in the infection characteristics in that trial (e.g., a higher proportion of *A. baumanii* infections) [2]. Indeed, this molecule is a good option, among the new β-lactams (NBLs), for the treatment of pneumonia caused by carbapenem-resistant Gram-negative bacilli [3,4].

Therefore, FDC is being used to fight Gram-negative bacilli with difficult-to-treat resistance (DTR), such as carbapenemase-metallo-β-lactamase-producing and other non-fermenting multidrug-resistant (MDR) bacilli with limited therapeutic choices [5]. In particular, some studies have validated FDC as a possible option for use against *Enterobacterales*, *Pseudomonas aeruginosa*, *Acinetobacter baumannii*, *Burkholderia* spp., *Stenotrophomonas maltophilia* and carbapenemase-producing *Klebsiella pneumoniae* (KPC) [5,6].

Different PK/PD studies reported the dosing and probability of the target attainment (PTA) of FDC in critical illness [7,8], but some conditions that deserve special consideration, including obesity, augmented renal clearance (ARC), renal replacement therapy (RRT) and ECMO, were not comprehensively considered [9,10].

Kobic et al. described a patient with pneumonia and a blood stream infection (BSI) caused by *P. aeruginosa*, considering the PD target of 82% *f*T > MIC (breakpoint MIC of 4 mg/L), and administered with a dosage of 2 g q8h (over 3 h) [11]. The achieved target was higher than 90% *f*T > MIC4 mg/L, and the therapy led to a clinical cure [12]. Konig et al. reported a case series of five patients affected by pneumonia and/or blood stream infections (BSIs): four were administered with 2 g q8h and one with 1 g q8h, all with 3 h infusions. Both schemes achieved both the targets of 100% *f*T > MIC ≤ 2 mg/L and *f*T > 4 × MIC ≤ 2 mg/L. Nevertheless, only 3/5 achieved microbiological cures, and 2/5 died. This suggests that, depending on the clinical context, the proposed PK/PD targets are questionable [13].

This issue is even more complex, considering that the FDC susceptibility breakpoints differ markedly across regulatory bodies: in detail, FDA indicates a susceptibility breakpoint of ≤1 mg/L, EUCAST of ≤2 mg/L, and CLSI of ≤4 mg/L [12,14,15]. Moreover, in some previous works it has been suggested that critically ill patients, particularly when infected with bacteria with borderline susceptibility, the pharmacokinetic/pharmacodynamic (PK/PD) targets may be increased to multiples of the MIC, such as a 4 × MIC [12,16]. The rationale for adopting a 4 × MIC threshold as an aggressive PK/PD target for beta-lactams in critically ill patients is supported by a growing body of evidence, including a recent systematic review and meta-analysis demonstrating that the attainment of a 100% fT > 4 × MIC was independently associated with higher clinical cure rates and a lower risk of microbiological failure in critically ill patients with Gram-negative infections [9,17]. In particular critical contexts, such as infections caused by pathogens at the upper boundary of susceptibility, even more stringent PD targets could be hypothesized and explored, such as 6 × MIC (*f*Cmin > 12 mg/L), based on the EUCAST breakpoint of 2 mg/L for Enterobacterales.

In this observational prospective case series, we aimed to study the relationship between PK/PD parameters, patient characteristics (obesity, renal function, infection site) and clinical and microbiological outcomes in a real-life clinical context.

## 2. Results

Ten patients with KP infections met inclusion criteria and were included in this analysis; the infection sites, bacterial isolates, FDC posology and RRT are reported in Table 1. Two patients received continuous veno-venous hemodialysis (CVVHD), and two received continuous venous hemofiltration (CVVH). The overall daily dose was significantly correlated with body weight (r = 0.755; *p* = 0.012).

The median age, BMI, eGFR (CKD-EPI formula), anthropometric and demographic characteristics, and clinical and microbiological outcomes, in the general group and stratified by BMI classes, are outlined in Table 2. All the enrolled patients were Caucasian.

PK/PD characteristics of the studied population, divided by BMI classes, are described in Table 3. Reported concentrations are intended as the estimated unbound plasma concentration. No statistically significant difference was highlighted for calculated PK/PD parameters between healthy-weight and non-healthy-weight groups.

### 2.1. Body Mass Index and Clinical Outcomes

Among the enrolled patients, an analysis of the impact of BMI on clinical outcomes was performed, merging the overweight and obese subgroups, due to the small sample size.

**Figure 1:**
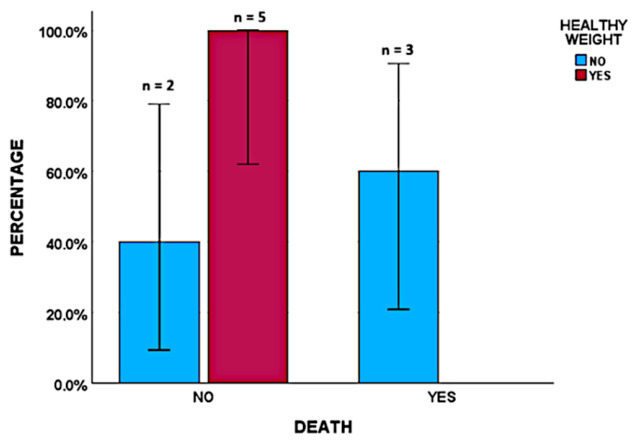
Relation between weight characterization and 30-day mortality (*p* = 0.038).

A significant association between weight groups and 30-day mortality was highlighted (χ^2^ = 4.286, *p* = 0.038), as illustrated in Figure 1. Notably, no deaths were observed among the healthy-weight group, while one (out of two cases, 50%) and two deaths (out of three cases, 66.6%) were observed in overweight and obese patients, respectively.

Similarly, a healthy BMI was positively associated with the microbiological outcome, with four out of five (80%) patients reaching eradication (χ^2^ = 6.667, *p* = 0.010). Strikingly, all overweight and obese patients failed to achieve microbiological eradication in the observation period.

### 2.2. PK/PD, Clinical and Microbiological Outcomes

**Figure 2:**
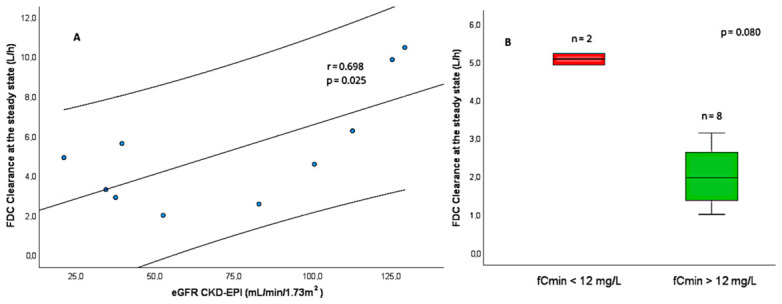
Association between eGFR and drug CLss (panel (**A**)) and the most aggressive PK/PD target attainment (6 × MIC EUCAST, 12 mg/L, panel (**B**)) (*p* = 0.08). FDC = cefiderocol; *f*C_min_ = free minimum drug concentration.

All patients reached the conventional PK/PD target of *f*C_min_ > 4 mg/L and the more aggressive target of *f*C_min_ > 4 × MIC (8 mg/L), while eight patients achieved the exploratory *f*C_min_ > 6 × MIC (12 mg/L) target. The two patients who did not reach the highest *f*C_min_ target had a *f*T > 12 mg/L of 78% and 98%. The patients’ weight was significantly and positively correlated with patients’ *f*C_min_ (r = 0.661; *p* = 0.038), suggesting that the weight-adjusted dose yielded a more than proportional increase in the plasma exposure. In fact, the patients’ weight was not significantly correlated with the Vd (r = 0.192; *p* = 0.594). In turn, the eGFR appeared to be significantly correlated with FDC CL_ss_ (r = 0.698; *p* = 0.025, Figure 2). Accordingly, a borderline relationship between FDC Cl_ss_ and the most aggressive PK/PD target attainment was observed (*p* = 0.08). Patients with a drug Cl_ss_ higher than 4.92 L/h did not reach the PK/PD target.

**Figure 3:**
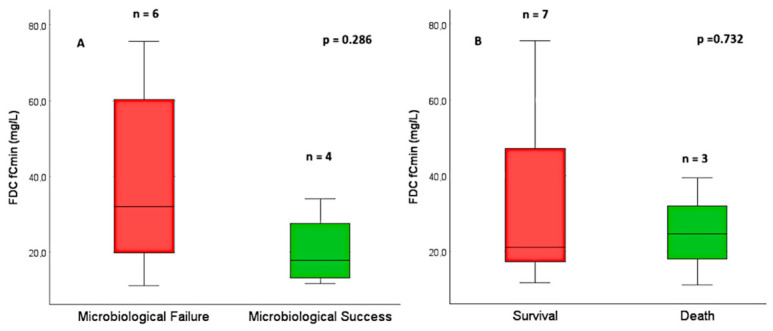
Differences in free FDC plasma minimum concentrations according to microbiological and clinical outcomes in terms of eradication (panel (**A**)) and survival at 30 days (panel (**B**)). FDC = cefiderocol; *f*C_min_ = minimum free drug concentration.

Nevertheless, no significant differences in PK or PK/PD and microbiological eradication or death/survival were observed, as shown in Figure 3.

**Figure 4:**
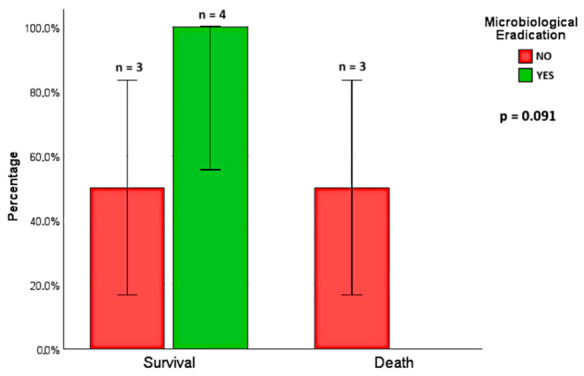
Association between patients’ survival and microbiological eradication. Whiskers indicate 95% error bars.

It is worth noting that the patients’ survival/death and microbiological eradication had only a borderline association (χ^2^ = 2.857, *p* = 0.091), as shown in Figure 4.

Finally, we tested the association between the infection site and the microbiological and clinical outcomes: patients with respiratory infections appeared slightly more likely to eradicate the infection (χ^2^ = 3.403, *p* = 0.063) and to survive (χ^2^ = 2.857, *p* = 0.091), compared to BSIs and abdominal infections.

## 3. Discussion

To date, the emergent issue of multidrug-resistant infections in ICUs, with the associated limited therapeutic choices, has increased the interest in the dose optimization of NBLs [18]. In fact, the recommended doses, based on PK/PD models, may not be adequate for ICU patients, particularly with concomitant conditions. Previous works investigated this issue associated with NBLs, all characterized by small sample sizes and heterogenicity [9,11,19].

The heterogeneity of the enrolled population, including variability in the infection site, renal replacement modality, administered dosing regimen, and resistance phenotype, constitutes a relevant limitation. These factors are recognized independent determinants of both PK/PD parameters and clinical outcomes, and their concurrent presence in a small cohort prevents any robust multivariate adjustment or causal inference regarding the independent contribution of BMI or drug exposure to treatment success. In 2021, Gatti et al. described 13 patients affected by pneumonia and/or BSIs treated with a FDC dosage of 2 g q8h and 1.5 g q8h (3 h infusion), and despite the fact that 77% reached quasi-optimal or optimal PK/PD targets (considering “quasi-optimal” a *f*C_min_ > 1 mg/L and “optimal” a *f*C_min_ above 4 mg/L), microbiological failure occurred in 54% of patients [9]. It appeared evident that other factors, irrespective of FDC concentrations, are involved in defining treatment success.

In this context, the aim of our study was to investigate possible associations between treatment outcomes, PK/PD perturbations and patients’ characteristics, particularly weight and renal function.

The apparently paradoxical finding of higher AUC and lower clearance levels in overweight patients alongside worse clinical outcomes is likely explained by several concurrent factors. First, the overweight subgroup comprised only two patients, making any group-level inference highly unreliable. Second, higher drug exposure in these patients also reflects the weight-based dosing rather than a true pharmacokinetic advantage, as FDC, like most beta-lactams, distributes primarily in extracellular water, generating a non-linear relationship between the weight-adjusted dose and the observed PK exposure. Third, and most importantly, BMI-associated comorbidities and greater baseline disease severity likely exerted a dominant effect on outcomes, overriding any potential benefit of the higher drug exposure.

In our study, the most significant data concerns the association between the BMI and outcome. All deaths (3/10) occurred in overweight or obese patients, while all healthy-weight patients survived (*p* = 0.038). Similarly, microbiological eradication was achieved in 80% of healthy-weight patients, while it was never achieved in overweight or obese patients (*p* = 0.036). The observed worst prognosis in overweight/obese patients was in contrast with the observed positive correlation between body weight and *f*C_min_ (r = 0.661): this is partially explained by the increased FDC dose in patients with higher body weight (r = 0.755; *p* = 0.012), while FDC, as many other beta-lactams, has a limited distribution in extracellular water. This hypothesis seems to be confirmed by the lack of a correlation between the weight and volume of distribution (r = 0.192; *p* = 0.594) in our patients.

The borderline association between FDC clearance and failure to reach the highest target (*p* = 0.08), with a critical threshold of CL_ss_ > 4.92 L/h, suggests that patients with preserved or increased renal function could benefit from higher doses or shorter dosing intervals in order to achieve the PK/PD targets. In fact, the significant correlation between the eGFR and CL_ss_ (r = 0.698; *p* = 0.025) confirms the predominant role of renal function in drug kinetics. In a PK/PD point of view, all patients reached the conventional targets (*f*C_min_ > 4 mg/L and > 4 × MIC), while the most aggressive target (100% *f*T > 6 × MIC) was achieved in 80% of cases.

Nevertheless, in this study, no statistically significant differences were observed between FDC kinetics between patients who achieved microbiological eradication or not, and, accordingly, no significant association was observed between PK/PD target attainment and both microbiological and clinical outcomes. This suggests that the main driver of microbiological and clinical outcomes in critical patients may be the clinical characteristics (e.g., body weight) and the infection site, while FDC PK could have a secondary role. The observed associations between BMI and outcomes, while statistically significant, should be interpreted cautiously in light of the small sample size and are intended to generate hypotheses for confirmation in larger, prospective studies.

This finding is in accordance with the previous evidence described by Gatti et al. [9]. This interpretation is further confirmed by the tendency to achieve better outcomes in patients with respiratory infections (eradication *p* = 0.063; survival *p* = 0.091) compared to those with BSIs or abdominal infections. This observation, despite not reaching statistical significance, is concordant with the good pulmonary penetration of FDC documented in the literature, as well as the different severities of patients’ clinical conditions at the baseline. Despite these observations, the results from this study have to be considered with caution, due to intrinsic limitations related to the exploratory nature of a monocentric case series, which limits the generalizability of the observed findings; the consequential small sample size, which limits our ability to overcome the masking effect of the different infection sites and resistance mechanisms (e.g., NDM vs KPC); and the absence of quantitative MIC determination, which could be useful for determining specific PK/PD targets for each patient.

## 4. Materials and Methods

This study was designed as an exploratory, hypothesis-generating investigation; no a priori sample size calculation was performed, and the findings should not be interpreted as statistically powered to confirm or exclude specific associations.

This was a prospective, single-center case series, designed to explore PK/PD parameters and their association with clinical and microbiological outcomes in critically ill patients receiving FDC.

### 4.1. Enrolled Patients

Patients with K. pneumoniae infections treated with FDC administered in 3 h infusion, who provided written informed consent, were enrolled. Exclusion criteria were pregnancy or age under 18 y.o. This study was performed in compliance with the Declaration of Helsinki and local review board regulations; all patients provided written informed consent, according to the local ethics committee standards (“Appropriatezza farmacologica della terapia anti-infettiva”, approved by Ethical Committee “A.O.U. CITTA’ DELLA SALUTE E DELLA SCIENZA DI TORINO—A.O. ORDINE MAURIZIANO DI TORINO—A.S.L. CITTÀ DI TORINO”, n° 456/2022). As microbiological and clinical endpoints, respectively, microbial eradication was evaluated after 14 days of treatment, while mortality was evaluated after 30 days.

The dosage of FDC in patients with obesity and renal insufficiency and in CRRT was determined by following the manufacturer’s indications [20,21].

Patients were stratified in 3 groups based on Body Mass Index (BMI): healthy weight (18.5–24.9); overweight (25–29.9); and obese (BMI of 30 or greater) [7].

Estimated GFR was calculated by CKD-EPI formula [22]: renal insufficiency was considered as an eGFR < 60 mL/min/1.73 m^2^.

### 4.2. Pharmacokinetic Analysis

Quantification of FDC was performed at the steady state (SS, after at least 5 days of treatment, according to the maximum half-life of 10 h in end-stage renal disease) before the infusion, at the end of infusion (3 h, C_max_) and then at 1 h; 3; and 5 h post-infusion. Steady-state sampling was performed after a minimum of 5–6 days of treatment, consistent with the pharmacokinetic profile of FDC in critically ill patients as reported in the literature [13,23].

FDC total plasma concentration was evaluated with a validated LC-MS/MS kit by CoQua lab (Turin, Italy) [24].

PK parameters were calculated using Phoenix WinNonLin^®^ software (ver. 32): free (*f*) concentrations were estimated considering a theoretical plasma protein binding of 58% [23].

PK/PD target of *f*C_min_ > MIC 4 mg/L (CLSI defined MIC) and more stringent targets of fC_min_ > 8 mg/L and *f*C_min_ > 12 mg/L (4 × and 6 × MIC 2 mg/L, EUCAST breakpoint for Enterobacterales, respectively) were considered [12,16]. The evaluated clinical outcomes were microbiological eradication and all causes of hospital 30-day mortality in hospital [9].

*f*AUC_0–8_ and drug clearance (Cl_ss_) were calculated through non-compartmental analysis (NCA) with a “linear up log down” fitting to describe the overall plasma exposure of FDC.

### 4.3. Statistical Analysis

IBM SPSS Statistics software 28.0 for Windows (Chicago, IL, USA) was used for statistical analysis. The Shapiro–Wilk test was used to test data for normal distribution. Non-normal variables were described as median values and interquartile range (IQR); categorical variables were described as numbers and percentages. Kruskal–Wallis and Mann–Whitney tests were used to test differences in continuous variables between groups considering the level of statistical significance (*p*-value) < 0.05. Correlations between continuous variables were tested with the Pearson test, while associations between categorical variables were tested through the chi-square test.

## 5. Conclusions

Overall, the results from this study suggest that FDC kinetics are mainly based on renal function, though the microbiological and clinical outcomes seemed to be mainly related to patients’ weight and clinical conditions, particularly related to the site of infection, overcoming the impact of PK/PD target attainment. In fact, the most conventional targets of *f*C_min_ above 4 mg/L and 8 mg/L were reached in all the patients. Our findings suggest an association between a higher BMI and worse microbiological and clinical outcomes in this small real-world cohort; however, given the observational design and limited sample size, no definitive causal inference can be drawn. These results should be regarded as preliminary and hypothesis-generating, warranting confirmation in larger, prospective, multicenter studies. These future studies should be adequately powered to investigate the different contributions of PK/PD target attainment and clinical/anthropometric characteristics to treatment success.

## Figures and Tables

**Table 1:** Demographic and clinical characteristics of enrolled patients. KPC NDM = New Delhi metallo-beta-lactamase-producing *Klebsiella pneumoniae*; BID = twice a day; TID = three times a day; QID = four times a day.

ID	Age/Sex	Weight	Infection	MDR Phenotype	Dose (mg and Dosing Interval)	RRT
**1**	74/M	84	Intra-abdominal infection	KP NDM	1500 q8h	Yes
**2**	67/M	70	Hospital-acquired pneumonia	KP NDM	1500 q8h	No
**3**	23/F	81	Blood stream infection	KP NDM	2000 q8h	No
**4**	73/M	88	Blood stream infection	KP NDM	2000 q8h	No
**5**	23/M	81	Blood stream infection	KP	2000 q8h	No
**6**	73/M	73	Respiratory infection in colonized patients	KP NDM	1500 q12h	Yes
**7**	73/M	73	Respiratory infection in colonized patients	KP NDM	1500 q8h	Yes
**8**	53/M	100	Blood stream infection	KP NDM	2000 q8h	No
**9**	56/M	75	Respiratory infection in colonized patients	KPC	1000 q8h	No
**10**	58/F	83	Blood stream infection	KP NDM	1500 q8h	Yes

**Table 2:** Characteristics of enrolled subjects. Continuous variables are described as mean values and interquartile range, IQR; dichotomic variables are reported as percentage (%). BMI = body mass index; PK/PD = pharmacokinetics/pharmacodynamics; Cr = creatinine; ClCr = creatinine clearance; ARC = augmented renal clearance; eGFR = estimated glomerular filtration rate; MIC = minimum inhibitory concentration.

Characteristics	General (n = 10)	Healthy Weight (n = 5)	Overweight (n = 2)	Obese (n = 3)	*p*-Value
Gender (male), n (%)	8 (80%)	5 (100%)	2 (100%)	1 (33.3%)	0.053
Weight (kg), median (IQR)	78.0 (72.3–85.0)	73.0 (68.5–74.0)	92.0 (84.0–/)	83.0 (81.0–/)	0.022
BMI (kg/m^2^), median (IQR)	26.8 (23.6–30.5)	23.8 (22.05–24.25)	29.3 (29.2–/)	31.3 (30.75–/)	0.022
Age, (years), median (IQR)	63 (46.7; 74)	68 (41; 74)	64.5 (54.0–/)	58 (41–66)	0.462
Renal replacement therapy, n (%)	4 (40%)	2 (40%)	1 (50%)	1 (50%)	0.933
Serum Cr level (mg/dL), median (IQR)	1.31 (0.68–1.90)	1.54 (0.77; 1.83)	1.53 (1.07–/)	0.6 (0.43-/)	0.624
eGFR < 60 mL/min/1.73 m^2^, n (%)	5 (50%)	3 (60%)	1 (50%)	1 (33.3%)	0.766
Death, n (%)	3 (30%)	0 (0%)	1 (50%)	2 (66.7%)	0.108
Microbiological eradication, n (%)	4 (40%)	4 (80%)	0 (0%)	0 (%)	0.036
CKD-EPI eGFR (mL/min/1.73 m^2^), median (IQR)	67.8 (36.7–115.9)	47 (37; 100)	55 (37–/)	124 (20–/)	0.696

**Table 3:** PK/PD parameters of considered patients stratified by BMI. *f*Conc = free concentration; K of elimination = elimination constant; *f*C_min_ = free minimum concentration; *f*C_max_ = free peak concentration; AUC_0–8_ = area under the concentration time curve during dosing interval; Cl_ss_ = clearance at the steady state; Vd = distribution volume; IQR = interquartile range.

PK Parameters	General (n = 10)	Healthy Weight (n = 5)	Overweight (n = 2)	Obese (n = 3)
*f*Conc_T0 (mg/L), mean (IQR)	23.49 (16.47–48.77)	22.39 (15.88; 45.02)	61.06 (48.43–/)	22.62 (11.95–/)
*f*Conc_T3 (mg/L), mean (IQR)	48.74 (37.13–58.85)	43.20 (32.11–61.72)	69.75 (55.52–/)	47.42 (37.17–/)
*f*Conc_T4 (mg/L), mean (IQR)	41.11 (29.80–55.33)	39.07 (28.89–57.04)	73.46 (52.80–/)	35.54 (23.79–/)
*f*Conc_T6 (mg/L), mean (IQR)	32.29 (22.45–56.54)	35.99 (23.42–56.85)	61.03 (40.89–/)	22.71 (12.40–/)
*f*Conc_T8 (mg/L), mean (IQR)	22.78 (13.71–44.52)	21.01 (13.01–47.10)	57.44 (39.29–/)	19.83 (9.81–/)
Half-life (h), mean (IQR)	4.70 (3.23–10.19)	4.78 (3.02; 11.8)	11.07 (9.36–/)	3.56 (3.12–/)
*f*C_max_ (mg/L), mean (IQR)	52.34 (37.13–62.37)	54.61 (32.11; 64.53)	74.81 (55.52–/)	47.42 (37.17–/)
*f*C_min_ (mg/L), mean (IQR)	21.50 (13.71–40.22)	21.01 (13.01; 38.49)	56.48 (39.29–/)	19.83 (9.81–/)
*f*AUC_0–8_ (h·mg/L), mean (IQR)	272.56 (211.20–426.67)	276.15 (197.72; 430.04)	520.90 (383.49–/)	257.36 (160.77–/)
PK/PD target achievement *f*C_min_ > 4 mg/L, n (%)	10 (100%)	5 (100%)	2 (100%)	3 (100%)
PK/PD target achievement *f*C_min_ > 8 mg/L (4 × MIC), n (%)	10 (100%)	5 (100%)	2 (100%)	3 (100%)
PK/PD target achievement 100%*f*T > 12 mg/L (6 × MIC), n (%)	8 (80%)	4 (80%)	2 (100%)	2 (66.7%)
Cl_ss_ (L/h), mean (IQR)	2.36 (1.4–3.6)	2.3 (1.2–3.9)	1.5 (1.3–/)	3.1 (2.4–/)
Vd (L), mean (IQR)	22.7 (14.5–23.9)	23.2 (9.6–25.3)	22.8 (22.2–/)	16.3 (16.1–/)

## Data Availability

Data are available upon request.
